# Body mass index and outcome in renal transplant recipients: a systematic review and meta-analysis

**DOI:** 10.1186/s12916-015-0340-5

**Published:** 2015-05-12

**Authors:** Jeffrey A Lafranca, Jan NM IJermans, Michiel GH Betjes, Frank JMF Dor

**Affiliations:** 1 grid.5645.2000000040459992XDepartment of Surgery, division of HPB & Transplant Surgery, Erasmus MC, University Medical Center Rotterdam, ‘s Gravendijkwal 230, PO BOX 2040, 3000 CA Rotterdam, The Netherlands; 2 grid.5645.2000000040459992XDepartment of Nephrology, Erasmus MC, University Medical Center Rotterdam, ‘s Gravendijkwal 230, PO BOX 2040, 3000 CA Rotterdam, The Netherlands

## Abstract

**Background:**

Whether overweight or obese end stage renal disease (ESRD) patients are suitable for renal transplantation (RT) is often debated. The objective of this review and meta-analysis was to systematically investigate the outcome of low versus high BMI recipients after RT.

**Methods:**

Comprehensive searches were conducted in MEDLINE OvidSP, Web of Science, Google Scholar, Embase, and CENTRAL (the Cochrane Library 2014, issue 8). We reviewed four major guidelines that are available regarding (potential) RT recipients. The methodology was in accordance with the Cochrane Handbook for Systematic Reviews of Interventions and written based on the PRISMA statement. The quality assessment of studies was performed by using the GRADE tool. A meta-analysis was performed using Review Manager 5.3. Random-effects models were used.

**Results:**

After identifying 5,526 studies addressing this topic, 56 studies were included. We extracted data for 37 outcome measures (including data of more than 209,000 RT recipients), of which 26 could be meta-analysed. The following outcome measures demonstrated significant differences in favour of low BMI (<30) recipients: mortality (RR = 1.52), delayed graft function (RR = 1.52), acute rejection (RR = 1.17), 1-, 2-, and 3-year graft survival (RR = 0.97, 0.95, and 0.97), 1-, 2-, and 3-year patient survival (RR = 0.99, 0.99, and 0.99), wound infection and dehiscence (RR = 3.13 and 4.85), NODAT (RR = 2.24), length of hospital stay (2.31 days), operation duration (0.77 hours), hypertension (RR = 1.35), and incisional hernia (RR = 2.72). However, patient survival expressed in hazard ratios was in significant favour of high BMI recipients. Differences in other outcome parameters were not significant.

**Conclusions:**

Several of the pooled outcome measurements show significant benefits for ‘low’ BMI (<30) recipients. Therefore, we postulate that ESRD patients with a BMI >30 preferably should lose weight prior to RT. If this cannot be achieved with common measures, in morbidly obese RT candidates, bariatric surgery could be considered.

**Electronic supplementary material:**

The online version of this article (doi:10.1186/s12916-015-0340-5) contains supplementary material, which is available to authorized users.

## Background

As the incidence of overweight and obesity rises globally, so does the number of end stage renal disease (ESRD) patients with obesity [1]. Renal transplantation (RT) is the preferred therapeutic option for ESRD, however, whether obese patients are suitable for RT is often debated due to the higher risk of complications [2]. Several guidelines state that obesity is not considered an absolute contra-indication, although patients with a body mass index (BMI) above 40 or 45 should not be considered for RT [3,4]. On the other hand, the guidelines state that if the transplant surgeon determines that the body composition of the potential RT recipient does not constitute an increased surgical risk, the patient should be suitable for RT. However, this does not take into account that it is not only the surgery itself that poses a possible risk. Equally important is the incidence of post-transplant complications in the obese recipient. Observational studies in the general population have demonstrated that obesity is an independent risk factor for chronic vascular disease [5]. Obesity is also associated with a number of risk factors for chronic vascular disease, including hypertension, dyslipidaemia, and diabetes [6]. Of note, in general, the most important mortality and morbidity post-transplant is due to cardiovascular complications [7].

Other possible complications that have previously been associated with a higher incidence in obese recipients are delayed graft function (DGF), impaired graft survival, longer hospital stay, higher costs, higher incidence of new onset of diabetes after transplantation (NODAT) and increased mortality [8-11]. Intuitively, all overweight potential recipients should lose weight prior to transplantation. Usually, dietary restriction is applied under the supervision of a dietician. However, in most cases, the desired result is not achieved, caused by several factors such as the need for dialysis three times a week, a low exercise tolerance, and comorbidities. In case of peritoneal dialysis, patients are known to increase in weight because the dialysate contains a high concentration of dextrose [12]. The body absorbs some of this dextrose during the dwell, which can lead to weight gain. Bariatric surgery (in case of morbid obesity or a BMI >35 with one or more comorbidities) could be considered, as it has proven to be successful in weight reduction in non-ESRD patients [13,14]. Few studies are available regarding bariatric surgery pre- or post-transplantation in (morbidly obese) ESRD-patients, however these all show promising results [15-17]. As has been recently published by Gill et al. [18], the transplant community needs to realize that even obese RT recipients have a significant survival benefit from transplantation despite the reduced risk of death of obese dialysis patients.

Recently, Nicoletto et al. [19] carried out a systematic review and meta-analysis on the same topic, and conclude that obese patients have an increased risk for DGF. However, they only included 21 studies and did not include surgical outcome in these patients, which is an important topic in our opinion, as patients are frequently declined for RT because of the increased risk for surgical complications.

The aim of the present systematic review and meta-analysis is to give a more in-depth insight in (metabolic, survival, and surgical) outcome of low (<30) versus high (>30) BMI recipients after RT.

## Methods

All aspects of the Cochrane Handbook for Interventional Systematic Reviews were followed [20], and the manuscript was written according to the PRISMA statement [21].

### Literature search strategy

Comprehensive searches were carried out in Embase, MEDLINE OvidSP, Web of Science, Google Scholar, CENTRAL (the Cochrane Library 2013, issue 5), and the Transplant Library. The search was performed for articles published until August 2014 relevant to outcome of kidney transplant recipients, both from a living or deceased donor. No language restriction was applied. Studies were included concerning patients that underwent RT, in which the recipients were divided according to BMI classification. As a cut-off value, a BMI of 30 was used to classify the included patients between ‘low’ (<30) and ‘high’ (>30) BMI, according to the definitions of the World Health Organization [22]. Included outcome measures were: mortality (defined as death within follow-up of each study), patient survival at years 1, 2, and 3, graft survival at years 1, 2, and 3, primary non-function, DGF (in 10 out of 30 studies defined as the need for dialysis within 7 days of transplantation), acute rejection, chronic rejection, graft loss, estimated glomerular filtration rate, operation duration, length of stay, lymphoceles, wound infection, incisional hernia, hematoma, wound dehiscence, surgical adverse events, NODAT, hypertension, and CMV infection. Search terms for each search-engine are provided as Additional file 1. Manual reference checks in included papers were performed to check for potentially missing studies.

### Guideline analysis

In addition to the literature search, we searched for guidelines regarding (potential) RT recipients in order to put the studies and their results in perspective. Specifically, sections about (pre-operative) overweight or obesity and RT suitability were reviewed.

### Literature screening

Studies were evaluated for inclusion by two independent researchers (JAL, FJMFD) for relevance to the subject. Study selection was accomplished through several phases of screening. First, studies were excluded if they were one of the following: case-reports, letters, editorials, case-series, animal studies, or if the abstract revealed no relevance to the subject. For publications without abstract, the full text was acquired. In the next phase, inclusion required that studies described two or more groups of RT recipients divided based on their BMI and described relevant outcome measures.

### Data extraction and critical appraisal

The level of evidence of each paper was established using the GRADE tool [23]. The GRADE approach defines the quality of a body of evidence by consideration of within study risk of bias (methodological quality), directness of evidence, heterogeneity, precision of effect estimates, and risk of publication bias.

### Statistical analysis

A meta-analysis was performed using Review Manager version 5.3 (The Nordic Cochrane Centre, Copenhagen, Denmark). Random-effects models were used to account for possible clinical heterogeneity. Depending on the outcome, results were presented in forest plots with risk ratios or mean differences. Overall effects were determined using the Z-test; 95% CIs of these values were given and *P* <0.05 was considered statistically significant. Heterogeneity between studies was assessed by three methods. First, a Tau^2^ test and a χ^2^ test were performed for statistical heterogeneity, with a *P* <0.1 being considered statistically significant. Also, *I*^*2*^ statistics were used to assess clinical heterogeneity, where an *I*^*2*^ of 0% to 40% is considered as low heterogeneity, 30% to 60% as moderate heterogeneity, 50% to 90% as substantial heterogeneity, and 75% to 100% as considerable heterogeneity. Where studies reported on two or more high or low BMI groups, pooled mean estimates and standard deviations were calculated. Group means were weighted by the number of recipients in each study group. Funnel plot analysis was used to assess possible publication bias.

## Results

We included four major guidelines that are currently available regarding (potential) RT recipients: the Kidney Disease Improving Global Outcomes (KDIGO) ‘Clinical Practice Guideline for the Care of the Kidney Transplant Recipient’ [24], ‘Assessment of the Potential Kidney Transplant Recipient’ (5^th^ edition, 2010) by the UK Renal Association [25], the ‘Guideline on Kidney Donor and Recipient Evaluation and Perioperative Care’ by the European Renal Best Practice (ERBP) guideline body [26], and Kidney Health Australia – Caring for Australasians with Renal Impairment (KHA-CARI): ‘Recipient Assessment for Transplantation’ and ‘Obesity in renal transplantation’ [27].

The KDIGO guidelines state that, in RT recipients, obesity is associated with cardiovascular events and mortality. Furthermore, they mention that there is little reason to believe that weight reduction measures are not equally effective in obese potential RT recipients as in the general population. However, there is some indication that pharmacological and surgical management of obesity may be more likely to cause harm than in the general population. They recommend that additional research is needed to determine the effect of bariatric surgery on outcomes in RT recipients.

The UK Renal Association guideline states that obese patients (BMI >30 kg/m^2^) present technical difficulties and are at increased risk of peri-operative complications. They should be screened rigorously for cardiovascular disease and each case should be considered individually. Although obesity is not an absolute contra-indication to transplantation, individuals with a BMI >40 kg/m2 are less likely to benefit from RT.

The ERBP guideline states that the association between BMI and patient survival after RT is controversial based on current literature. Furthermore, it is recommended that RT candidates with a BMI >30 kg/m2 should lose weight prior to RT.

The KHA-CARI guidelines recommend that obesity alone should not preclude a patient from being considered for RT. Furthermore, they state that as a pre-transplant BMI >40 kg/m2 may not be associated with a survival advantage compared to remaining on dialysis, the suitability for transplant should be carefully assessed on an individual basis. Lastly, as patient and graft survival of obese transplant recipients may be mediated by comorbid factors, particularly cardiovascular, they recommend screening of obese transplant candidates for cardiovascular disease.

### Literature search results

**Figure 1:**
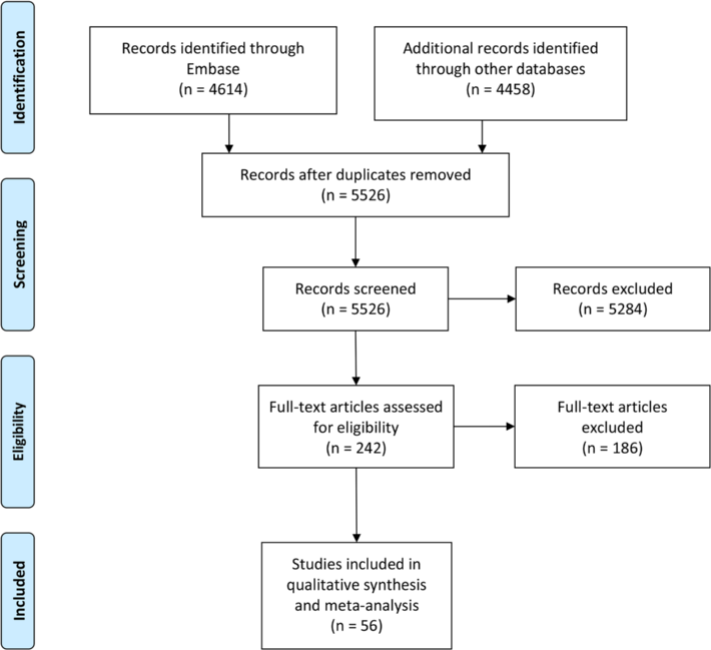
PRISMA (Preferred Reporting Items for Systematic Reviews and Meta-Analyses) flowchart of the systematic literature search.

**Figure 2:**
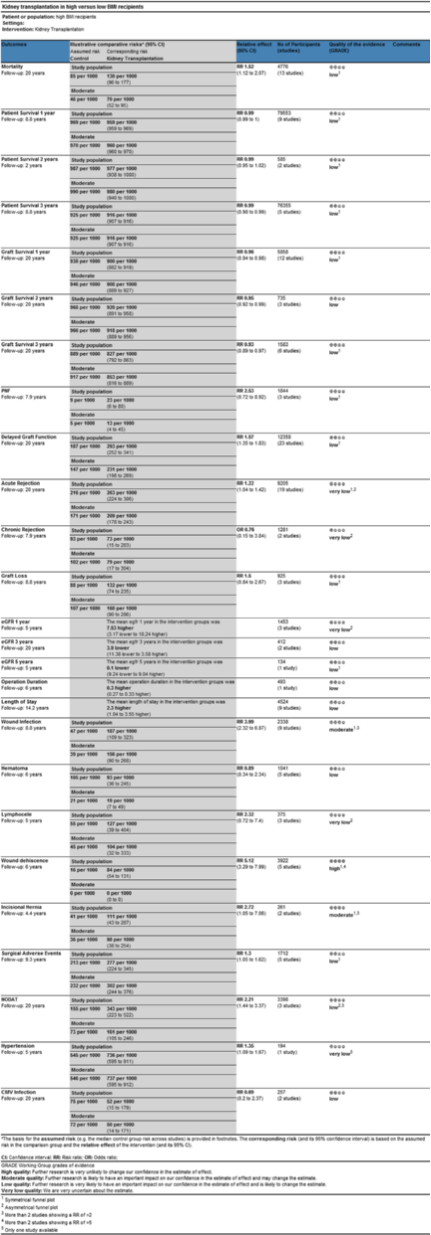
Summary of findings table of extended criteria in live kidney donation generated by the GRADE tool.

Out of 5,526 unique papers identified in the initial search, 56 studies were included. The PRISMA flow diagram for systematic reviews is presented in Figure 1. Data for 37 outcome measures were extracted (representing data of more than 209,000 recipients) of which 26 could be meta-analyzed. The characteristics of the included studies are presented in Table 1. The assessment of the quality of the included studies is presented in Figure 2.

**Table 1:** **Overview of the included studies in the systematic review**

**Reference**	**Country**	**Year**	**BMI groups**	**n**
Aalten [9]	The Netherlands	2006	<30	1,871
			≥30	196
Abou-Jaoude [28]	Lebanon	2010	<18.5	10
			18.5–24.9	62
			25.0–29.9	47
			≥30	16
Bardonnaud [29]	France	2012	<30	179
			≥30	21
Begov [30]	USA	2013	18–24.9	189
			25–29.9	169
			30–34.9	110
			≥35	78
Bennett [31]	USA	2011	<30	439
			30.1–34.9	109
			>35	89
Cannon [2]	USA	2013	<30	52,668
			30.0–34.9	15,010
			35.0–39.9	5,744
			≥40	1,561
Chang [32]	Australia	2007	<18.5	218
			18.5–24.9	2,719
			25.0–29.9	1,880
			≥30	867
Chow [33]	China	2006	<25	113
			≥25	37
Cockbain [34]	UK	2012	<18.5	731
			18.5–24.9	(Combined)
			25.0–29.9	421
			≥30	197
Curran [35]	USA	2014	<20	100
			20–24.9	429
			25–29.9	364
			30–34.9	184
			≥35	74
Ditonno [36]	Italy	2011	<18.5	68
			18.6–24.9	310
			25.0–29.9	143
			30–34.9	32
			≤35	10
Dobbels [37]	Belgium	2008	<18.5	2,156
			18.6–24.9	18,345
			25.0–29.9	15,040
			30–34.9	7,520
			≤35	3,401
Espejo [1]	Spain	2003	<30	40
			≥30	40
Farooq [38]	USA	2014	<36	27
			≥36	27
Furriel [39]	Portugal	2011	18.5–24.9	295
			25.0–29.9	127
			≥30	26
Gill [40]	USA	1993	<27	85
			>30	85
Gill [41]	USA	2013		Not reported
Gore [8]	USA	2005	<18.5	1,042
			18.5–24.9	12,089
			25.0–29.9	8,765
			30–34.9	3,891
			≥35	1,590
Grosso [42]	Italy	2012	<25	122
			25–30	190
			>30	64
Gusukuma [43]	Brazil	2011	<30	2,822
			30–34.9	185
			35–39.9	43
			≥40	4
Halme [44]	Finland	1995	20–25	235
			>30	47
Holley [45]	USA	1990	≤27 (male)	50
			≤25 (female)	
			≥30	46
Howard [46]	USA	2002	<25	457
			25–29.9	278
			≥30	98
Impedovo [47]	Italy	2012	<18.5	98
			18.6–24.9	428
			25–29.9	179
			≥30	58
Johnson [48]	Australia	2002	≤30	434
			>30	59
Kamali [49]	Iran	2010	<30	146
			>30	34
Karabicak [50]	USA	2011	<20	74
			20–24.9	215
			25–29.9	193
			30–34.9	99
			≥35	61
Lynch [51]	USA	2009	<20	33
			20–30	491
			≥30	345
McGee [52]	USA	2008	<25	Not reported
			25–29	
			≥30	
Marcen [53]	Spain	2007	<18.5	63
			18.5–24.9	617
			25–29.9	255
			≥30	65
Marks [54]	USA	2004	≤28	224
			≥35	23
Massarweh [55]	USA	2005	<30	137
			≥30	56
Mehta [56]	USA	2007	<30	37
			≥30	16
Meier-Kriesche [10]	USA	1999	≤25	240
			>25	165
Modlin [57]	USA	1997	<27	127
			>30	127
Molnar [58]	USA	2011	≤19.9	Not reported
			20–21.99	
			22–24.99	
			25–29.99	
			30–34.99	
			≥35	
Moreira [59]	Brazil	2013	<18.5	31
			18.5–24.9	248
			25–29.9	120
			≥30	48
Papalia [60]	Italy	2010	18.5–25	110
			25–30	84
Patel [61]	USA	2011	<30	315
			≥30	160
Pieloch [62]	USA	2014	18.5–24.9	24,077
			35–40	6,055
Pirsch [63]	USA	1995	<27.5	466
			27.5–30	59
			>30	59
Powers [64]	USA	2010	<25	34
			25–30	34
			>30	20
Rais-Jalali [65]	Iran	2005	<20	56
			20–24.9	86
			25–29.9	28
			≥30	12
Rajab [66]	USA	2007	<25	411
			25–29.9	416
			30–34.9	292
			35–39.9	106
			>40	80
Ravindra [67]	USA	2013	<25	Not reported
			25–29	
			30–34	
			≥35	
Schwarznau [68]	USA	2008	<30	56
			>30	25
Singh [69]	Canada	2005	≤30	35
			>30	33
Tang [70]	USA	2011	18–24.9	136
			25–29.9	116
			30–34.9	66
			≥35	54
Tremblay [71]	USA	2014	<20	32
			20–24.9	119
			25–29.9	149
			30–34.9	110
			≥35	57
Turner [4]	USA	2007	<30	753
			≥30	241
Walczak [72]	USA	2010	<30	61
			>30	46
Weissenbacher [73]	Austria	2012	<25	746
			>25	367
Wolyniec [74]	Poland	2011	<30	29
			≥30	29
Yamamoto [75]	USA	2002	<30	28
			>30	28
Zaydfudim [76]	USA	2010	18.5–24.9	154
			25–29.9	192
			30–34.9	80
			≥35	38
Zrim [77]	Australia	2012	<18.5	10
			18.5–24.9	182
			25–29.9	194
			30–34.9	93
			≥35	29

Although the search could have identified randomized controlled trials, only observational studies were found, as randomized controlled trials addressing this topic do not seem to be feasible.

#### Survival outcome parameters

##### Mortality

**Figure 3:**
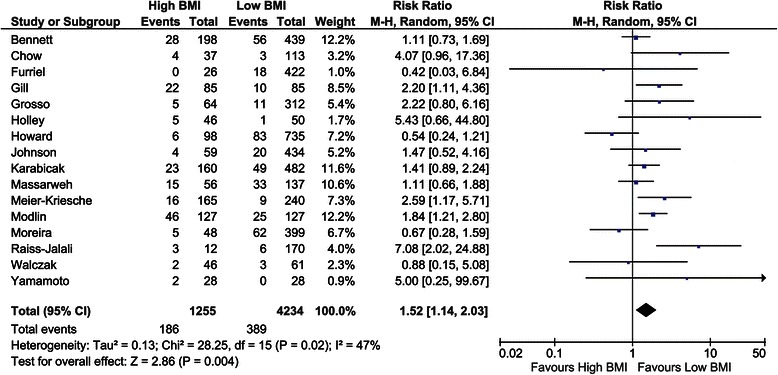
Forest plot of comparison: high versus low BMI recipients; outcome: mortality.

The number of deceased patients was studied in 16 studies including a total of 5,489 RT recipients [10,31,33,39,40,42,45,46,48,50,55,57,59,65,72,75]. The overall risk ratio was 1.52 (confidence interval (CI), 1.14–2.03; *P* = 0.004, *I*^*2*^ = 47%; *P* = 0.02) for high BMI recipients (Figure 3). Five studies assessed the mortality rate in a regression model [9,32,41,42,62]. Overall, there were no significant differences with an overall hazard ratio of 1.01 (CI, 0.89–1.15; *P* = 0.87, *I*^*2*^ = 87%; *P* <0.01). Massarweh et al. [55] also expressed mortality in odds ratios; OR, 1.39 (CI, 0.43–4.49; *P* = 0.58, *I*^*2*^ not applicable).

##### Patient survival (1-, 2-, and 3-year)

**Figure 4:**
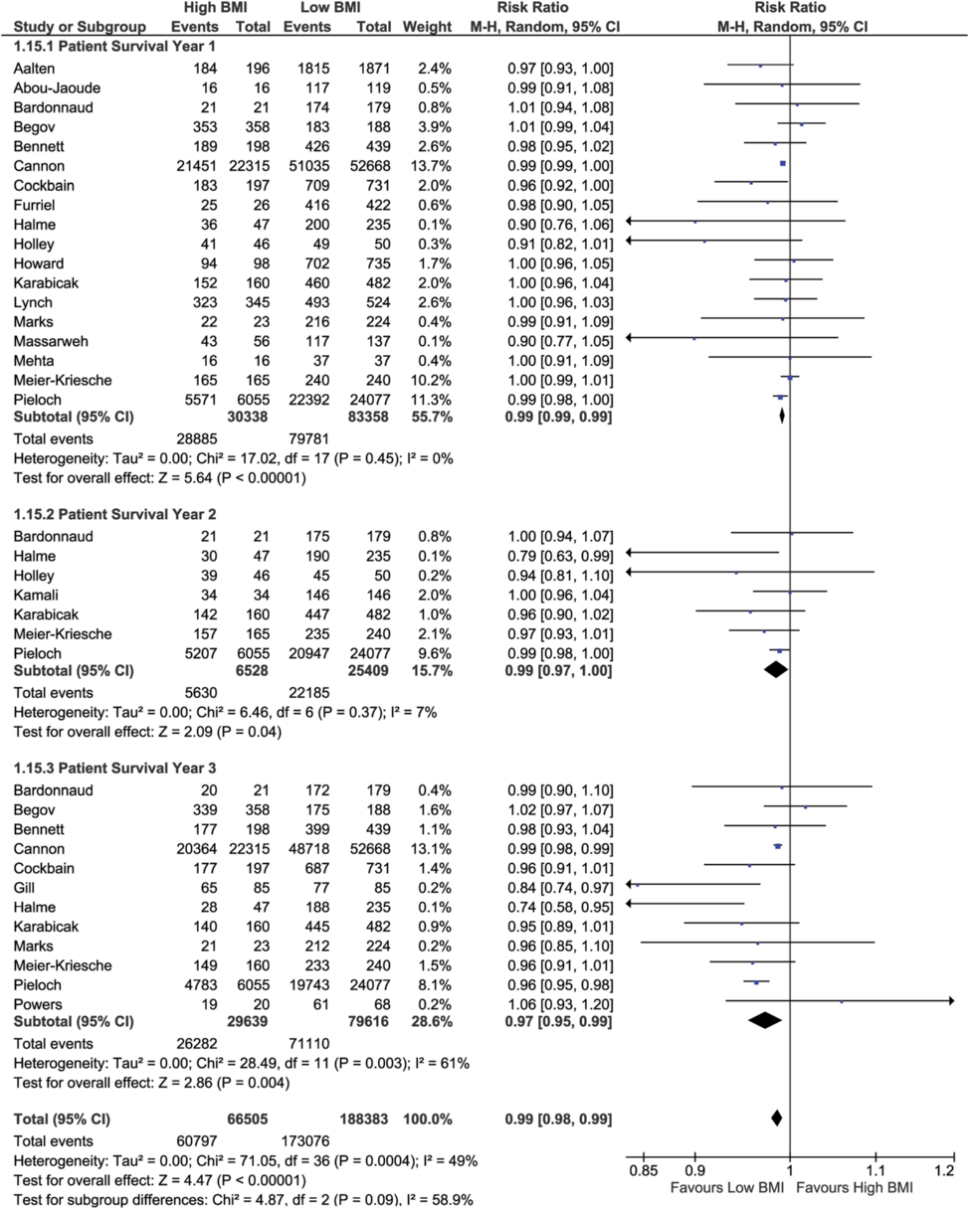
Forest plot of comparison: high versus low BMI recipients; outcome: patient survival at 1, 2, and 3 years.

One-year patient survival was analysed in 18 studies and showed better survival for low BMI recipients (risk ratio (RR) = 0.99, CI, 0.99–0.99; *P* <0.001, *I*^*2*^ = 0%; *P* = 0.45) [2,9,10,28-31,34,39,44-46,50,51,54-56,62]. At 2 years, seven studies showed a significant difference between recipient groups, again in favour of low BMI recipients (RR = 0.99, CI, 0.97–1.00; *P* = 0.04, *I*^*2*^ = 7%; *P* = 0.37) [10,29,44,45,50,62,78]. The 3-year patient survival was investigated in 12 studies, showing significant differences with a risk ratio of 0.97 (CI, 0.95–0.99; *P* = 0.004, *I*^*2*^ = 61%; P = 0.003; Figure 4) [2,10,29-31,34,40,44,50,54,62,64]. Interestingly, the five studies that included BMI in regression analyses showed that a higher BMI is associated with a higher patient survival with an overall hazard ratio of 0.93 (CI, 0.89–0.97; *P* <0.001, *I*^*2*^ 0%; *P* = 0.68) [2,29,30,51,53]. Grosso et al. [42] calculated an odds ratio of 27.98 (CI, 3.25–240.89; *P* = 0.002, *I*^*2*^ not applicable) in high BMI recipients.

##### Graft survival (1-, 2-, and 3-year)

**Figure 5:**
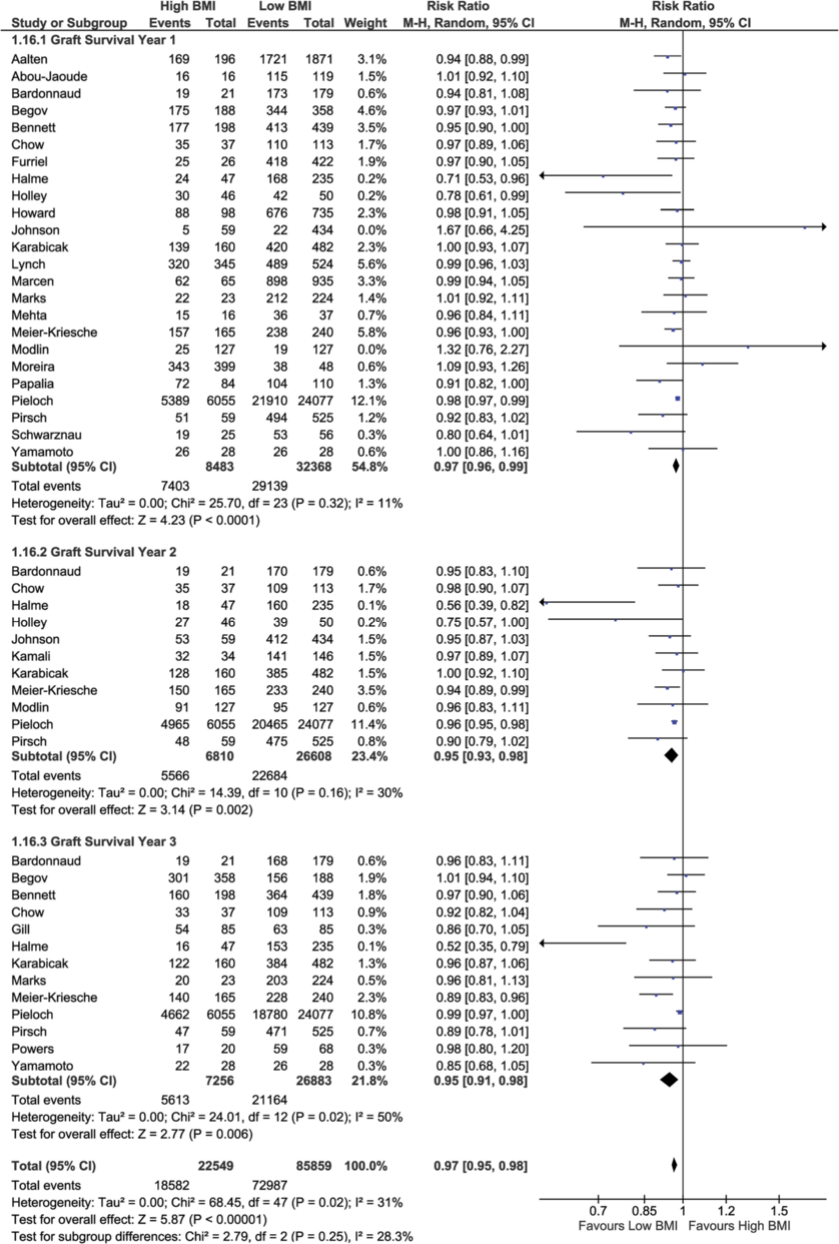
Forest plot of comparison: high versus low BMI recipients; outcome: graft survival at 1, 2, and 3 years and hazard ratio of graft survival.

Twenty-four studies investigated 1-year graft survival and showed a better graft survival in recipients with a low BMI (RR = 0.97, CI, 0.96–0.99; *P* <0.001, *I*^*2*^ = 11%; *P* = 0.32) [9,10,28-31,33,39,44-46,48,50,51,53,54,56,57,59,60,62,63,68,75]. Eleven studies assessed the 2-year graft survival [10,25,33,44,45,48,50,57,62,63,78]. The overall risk ratio was 0.95 (CI, 0.93–0.98; *P* = 0.002, *I*^*2*^ = 30%; *P* = 0.16). The 13 studies that analysed 3-year graft survival showed an overall risk ratio of 0.95 (CI, 0.91–0.98; *P* = 0.006, *I*^*2*^ = 50%; *P* = 0.02) [10,29-31,33,40,44,50,54,62-64,75]. In each year studied, graft survival was in favour of low BMI recipients (Figure 5). Seven studies included BMI as a parameter in regression analyses showing no significant relation between BMI and graft survival. The overall hazard ratio was 1.00 (CI, 0.96–1.04; *P* = 0.98, *I*^*2*^ 54%; *P* = 0.04) [2,9,29,30,48,52,53]. Grosso et al. [42] calculated an odds ratio (OR = 0.98, CI, 0.13–7.39; *P* = 0.98, *I*^*2*^ not applicable).

#### Kidney function outcome parameters

##### Delayed graft function

**Figure 6:**
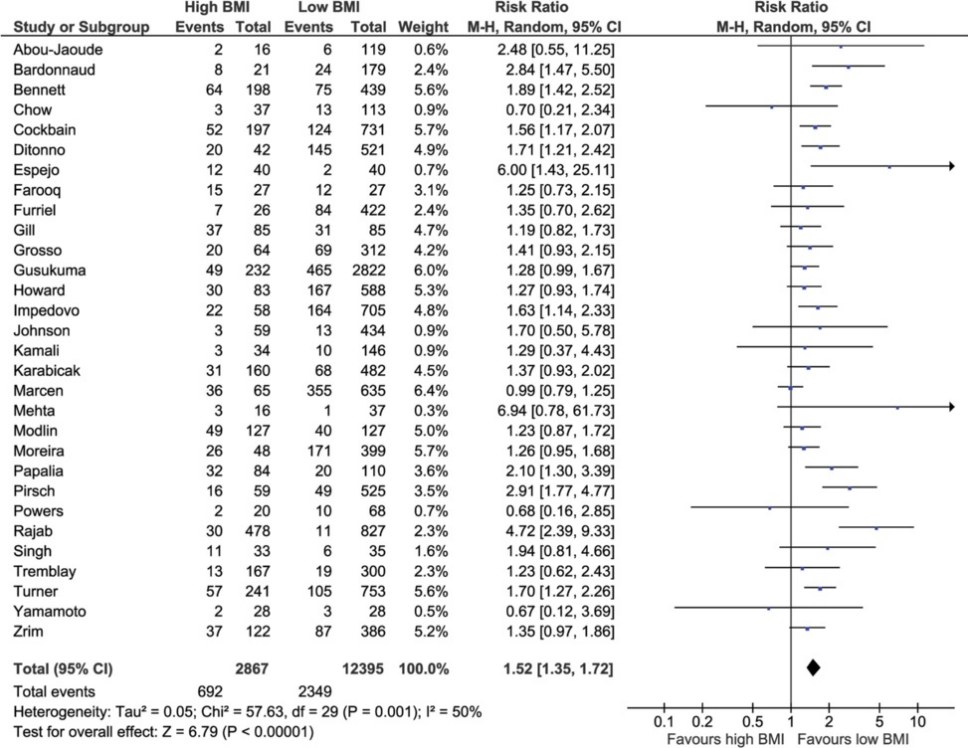
Forest plot of comparison: high versus low BMI recipients; outcome: delayed graft function.

The incidence of DGF was assessed in 30 studies encompassing a total of 15,262 recipients [1,4,28,29,31,33,34,36,38-40,42,43,46-48,50,53,56,57,59,60,64,66,69,71,75,77,78]. The overall risk ratio was 1.52 (CI, 1.35–1.72; *P* <0.001, *I*^*2*^ = 50%; *P* = 0.001; Figure 6). Six studies assessed DGF in ORs using a BMI of 30 as cut-off value [2,8,32,35,58,73]. The overall OR when pooling these studies was 1.38 (CI, 1.20–1.59; *P* <0.001, *I*^*2*^ = 92%; *P* <0.01). The pooled OR when using a BMI of 35 as a cut-off was 1.96 (CI, 1.69–2.28; *P* <0.001, *I*^*2*^ = 32%; *P* = 0.23) [8,35,58].

##### Acute rejection

**Figure 7:**
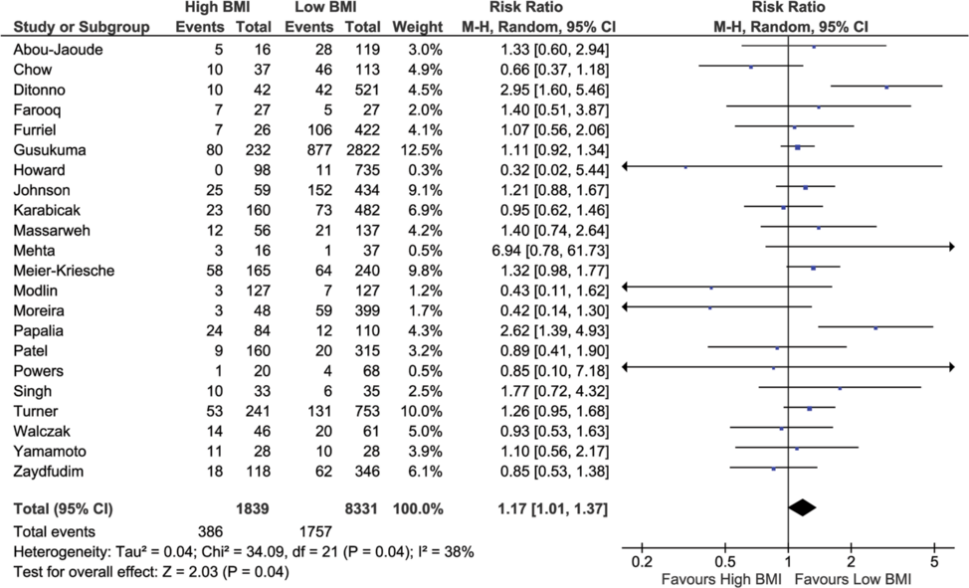
Forest plot of comparison: high versus low BMI recipients; outcome: acute rejection.

The incidence of acute rejection was investigated in 22 studies [4,10,28,33,36,38,39,43,46,48,50,55-57,59-61,64,69,72,75,76]. Twelve studies showed a lower risk ratio on acute rejection in low BMI recipients. The overall risk ratio, including 10,170 recipients, was 1.17 (CI, 1.01–1.37; *P* = 0.04, *I*^*2*^ = 38%; *P* = 0.04; Figure 7). Gore et al. [8] assessed the incidence of acute rejection in OR as 1.19 (CI, 1.11–1.28; *P* <0.001, *I*^*2*^ not applicable).

Other outcome parameters showing no significant differences in kidney function outcome parameters are outlined in Table 2.

**Table 2:** **Outcome parameters with no significant differences**

**Outcome parameter**	**Studies**	**RR (CI)**	***P*** **value**
Primary non function	3 [36,39,46]	2.53 (0.72–8.92)	0.15
Chronic rejection	2 [39,46]	0.18–3.54	0.76
Graft loss	5 [42,48,51,59,75]	1.14 (0.87–1.50)	0.34
		**Mean difference (CI)**	
eGFR year 1	3 [4,28,70]	7.53 mL/min (-3.17–18.24)	0.17
eGFR year 3	2 [33,70]	-3.90 mL/min (-11.38–3.58)	0.31
eGFR year 5	1 [70]	-0.10 mL/min (-0.24–9.04)	0.98

eGFR, Estimated glomerular filtration rate; RR, Risk ratio; CI, Confidence interval.

#### Surgical outcome parameters

##### Operation duration

**Figure 8:**
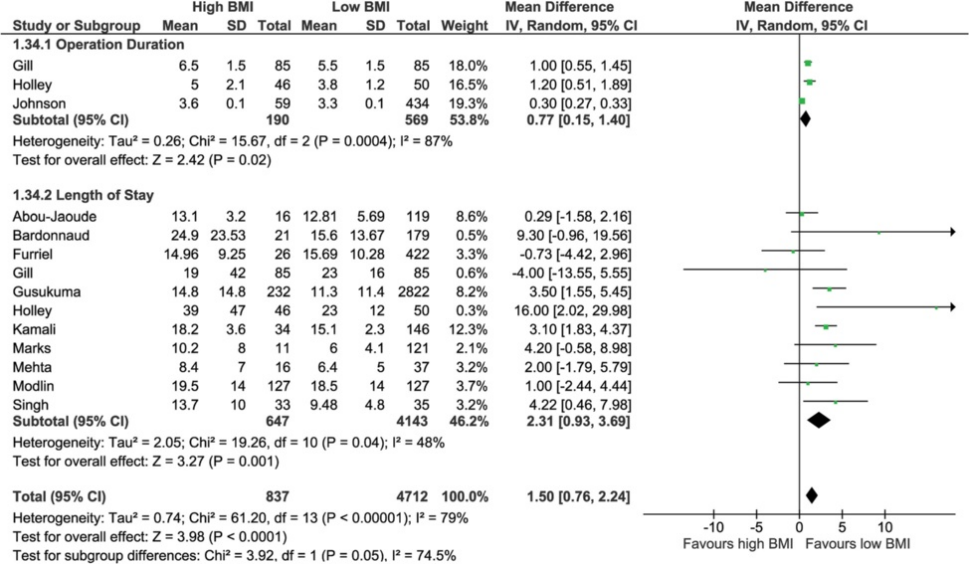
Forest plot of comparison: high versus low BMI recipients; outcome: operation duration and length of stay.

Only three studies investigated the operation duration in low versus high BMI recipients, showing a mean difference of 0.77 hours (CI, 0.15–1.40), with a statistically significant difference (*P* = 0.02, *I*^*2*^ = 87%; *P* <0.01; Figure 8) [40,45,48].

##### Length of stay

The length of hospital stay was assessed in 11 studies [28,29,39,40,43,45,54,56,57,69,78]. All studies but two showed a mean length of stay in favour of low BMI recipients [39,40]. The overall mean difference was 2.31 days (CI, 0.93–3.69; *P* = 0.001, *I*^*2*^ = 48%, *P* = 0.04; Figure 8).

##### Wound infection

**Figure 9:**
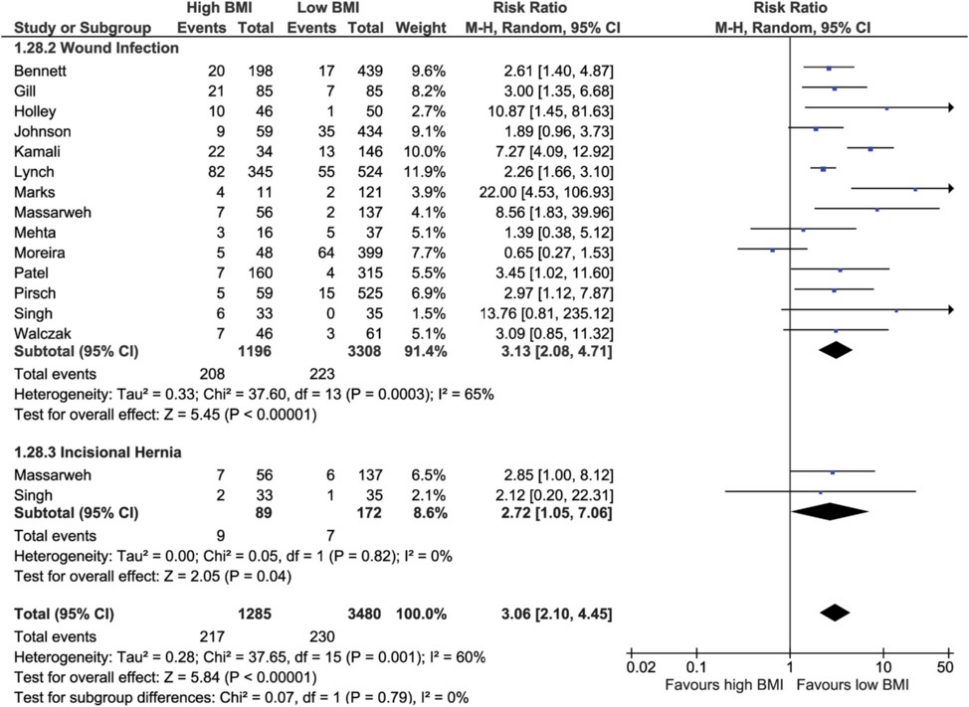
Forest plot of comparison: high versus low BMI recipients; outcome: wound infection and incisional hernia.

The incidence of wound infections was studied in 13 studies with a total of 4,504 recipients [31,40,45,48,51,54-56,59,61,63,69,72,78]. The overall risk ratio of this outcome was 3.13 (CI, 2.08–4.71; *P* <0.001, *I*^*2*^ = 65%; *P* <0.01; Figure 9).

##### Incisional hernia

Two studies assessed the incidence of incisional hernias [55,69]. The overall risk ratio was 2.72 (CI, 1.05–7.06; *P* = 0.04, *I*^*2*^ = 0%; *P* = 0.82; Figure 9).

##### Wound dehiscence

**Figure 10:**
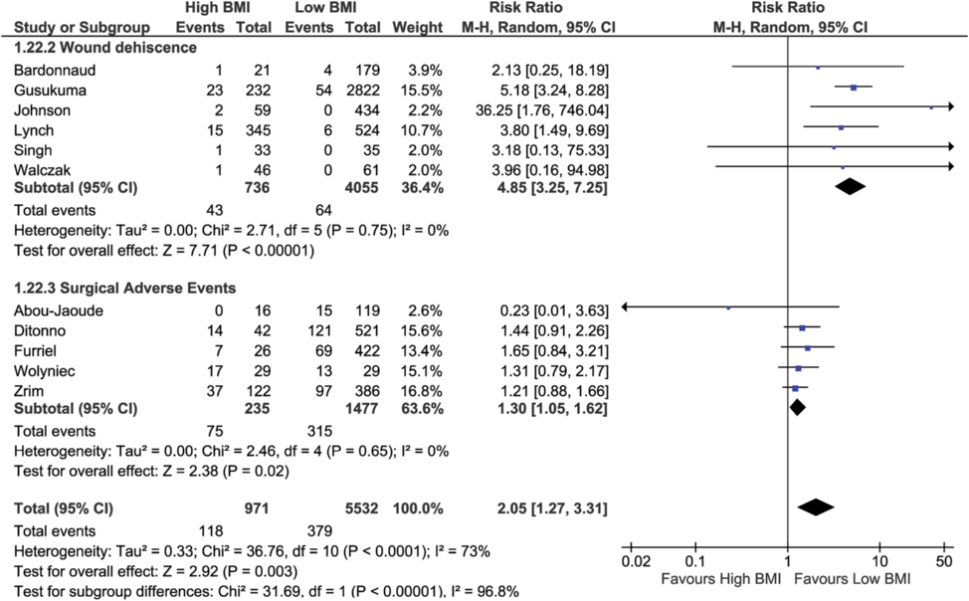
Forest plot of comparison: high versus low BMI recipients; outcome: wound dehiscence and surgical adverse events.

Six studies reported the incidence of wound dehiscence including 3,922 recipients [29,43,48,51,69,72]. The overall risk ratio was 4.85 (CI, 3.25–7.25; *P* <0.001, *I*^*2*^ = 0%; *P* = 0.75; Figure 10).

##### Surgical adverse events

Five studies investigated surgical adverse events, such as urologic, vascular, and haemorrhagic complications [28,36,39,74,77]. The overall risk ratio was 1.30 (CI, 1.05–1.62; *P* = 0.02, *I*^*2*^ = 0%; *P* = 0.65; Figure 10).

Other outcome parameters showing no significant differences in surgical outcome parameters are outlined in Table 3.

**Table 3:** **Outcome parameters with no significant differences**

**Outcome parameter**	**Studies**	**RR (CI)**	***P*** **value**
Lymphoceles	4 [29,40,69,72]	1.74 (0.74–4.11)	0.20
Hematoma	5 [48,55,69,72,78]	0.89 (0.34–2.34)	0.82

RR, Risk ratio; CI, Confidence interval.

#### Metabolic outcome parameters

##### NODAT

Six studies including 4,111 recipients investigated the incidence of new onset diabetes after transplantation [33,40,43,45,59,60]. Overall, a risk ratio of 2.24 (CI, 1.46–3.45; *P* <0.001, *I*^*2*^ = 53%; *P* = 0.06) was found.

##### Hypertension

Only one study assessed the incidence of hypertension in different BMI groups including 194 patients [60]. High BMI recipients had a higher risk on hypertension with a risk ratio of 1.35 (CI, 1.09–1.67; *P* = 0.005, *I*^*2*^ not applicable).

#### Other outcome parameters

##### Cytomegalovirus (CMV) infection

The incidence of CMV infection was addressed in two studies [33,72]. Overall, the risk ratio was 0.69 (CI, 0.20–2.37; *P* = 0.56, *I*^*2*^ = 14%; *P* = 0.28) in favour of low BMI recipients.

## Discussion

With this meta-analysis, we aimed to determine whether guidelines or policy should be revised with respect to suitability for RT of overweight and obese (potential) recipients, since this is often debated. There are several central questions behind this need for additional insight. Should obese ESRD-patients be transplanted at all? Are we and are these patients aware of all possible risks? Should we emphasize the need for weight loss, or even advise bariatric surgery before RT, and to whom?

The worldwide prevalence of obesity is rising, leading to an increasing number of patients with cardiovascular comorbidity, diabetes (metabolic syndrome) and, consequently, ESRD [79-81]. As RT is the golden standard in treating these patients, a good understanding of the consequences of transplanting overweight and obese ESRD-patients is needed. Several reviews have been written regarding this topic [82-93]. The KDIGO-guidelines state that observational studies report an association between obesity and mortality in RT recipients. The present study is the first meta-analysis investigating several (metabolic, survival, and surgical) outcome measures, and pooling data from a large number of studies (n = 56, including over 209,000 recipients).

Nicoletto et al. [19] recently published a systematic review and meta-analysis regarding this very subject. Their main finding was that recipient obesity is associated with an increased rate of DGF and that there was no association between obesity and acute rejection. One of the limitations of their study is that they included only 21 studies, whereas we included 56 publications. This could be explained by the fact that fewer databases have been searched by the authors (MEDLINE, EMBASE, and the Cochrane Library) than we did (Web of Science, Google Scholar, and the Transplant Library). Furthermore, the authors did not describe if any study was excluded based on the quality assessment of the Newcastle-Ottawa Quality Assessment Scale or the GRADE tool. Interestingly, the authors observed that studies published after 2003 show no differences in survival between BMI groups. Although they state that 2003 was used as a cut-off because of the fact that included patients were transplanted before 2000, they do not provide an explanation as to why obesity would pose a problem before 2000. In our opinion, other factors may contribute to this result, such as the fact that live kidney donation has increased over the years, providing better quality grafts resulting in increased graft and, thus, patient survival. Moreover, they did not analyse surgical outcome measures as wound infection and dehiscence. In our opinion these are important outcomes that should also be included in the informed consent procedure for the recipients. Finally, we have included a meta-analysis of hazard ratios of graft and patient survival in the included studies, showing more clearly that the BMI itself may not be the cause of worse outcome in RT recipients but rather other comorbidities associated with obesity such as diabetes or (cardio-)vascular disease. Perhaps different lifestyle recommendations should be provided to patients who remain on dialysis versus those who will be transplanted [94].

Our results clearly show that, in recipients with a higher BMI, graft and patient survival are worse, at least up to 3 years after transplantation. Interestingly, in regression analyses, regarding patient survival, having a higher BMI seems to be associated with a higher patient survival, and regarding graft survival there appears to be no significant relation with the BMI. This could be explained by the ‘obesity paradox’, an interesting phenomenon that has been described for haemodialysis patients [93], suggesting that patients on haemodialysis with a higher BMI tend to have an improved survival benefit. However, the improved survival benefit is associated with higher costs, more complications, and worse outcome after transplantation [95]. On the other hand, and perhaps most importantly, obese RT recipients still demonstrate significant survival benefit from transplantation compared to dialysis [18].

The kidney function outcome parameters show that the incidence of DGF and acute rejection is higher in high BMI recipients. A possible explanation is that the operation duration is longer in recipients with a higher BMI, which in itself is associated with higher DGF-rates [96]. The increased incidence of acute rejection might be explained by the fact that obesity is linked to inflammation and modified immune responses, potentially impacting allorecognition and alloimmunity [97]. Another possibility is the increased finding of (not clinically relevant) rejection because of the higher incidence of biopsies in case of DGF.

Regarding the metabolic outcome parameters, increasing BMI shows a significant correlation with the development of NODAT and hypertension, which is not surprising, knowing that overweight and obesity are common risk factors for developing these comorbidities [92,98].

All surgical outcome measures are significantly in favour of recipients with a low BMI, with exception of the incidence of hematoma and lymphoceles. A possible explanation could be that the latter two complications are not necessarily influenced by overweight or body composition, in contrast to wound dehiscence or hernias [99,100].

Although a large part of our systematic review concerns long-term outcome measures, we should bear in mind that the perioperative (surgical) outcome measures are of great importance. Many RT candidates with a high BMI are declined because the concern of possible surgical difficulties and inherent complications. As confirmed by the results of the meta-analysis, this concern is justified. Therefore, high BMI RT candidates should be referred to tertiary referral centres to centralise knowledge about and experience with this patient category, especially on a transplant surgical level. Additionally, it is another motivator to encourage RT candidates to lose weight prior to transplantation, ideally several years before the operation. Nephrologists can play a crucial and proactive role in this process.

In summary, we conclude that obesity prior to RT leads to impaired outcome after RT. Losing weight prior to transplantation might be of great importance, although it is unclear whether this is advantageous for ESRD patients who remain dependent on dialysis [101]. However, one should bear in mind that, even if sufficient weight loss cannot be achieved before transplantation, transplantation still leads to enormous advantages in terms of survival, health, and quality of life [18]. We have recently performed a study showing that patients who are deemed unsuitable for RT because of high BMI in one centre have excellent outcome when transplanted in a tertiary high-volume centre (Glijn et al., manuscript in preparation). For overweight or obese recipients that will be transplanted, conventional methods to lose weight, such as dietary advice, might not lead to the desired (or sufficient) effect [16,46,102]. Even though some weight loss might be achieved, after transplantation, the weight is often regained, possibly caused by the metabolic changes that may result in better nutrient absorption and/or reduced energy expenditure with improved renal function after transplantation. Furthermore, the increased quality of life may lead to a larger food intake [94,103].

Another, more effective, method to lose weight prior to or post-transplantation is bariatric surgery. Some studies have already been performed showing promising results [15-17,104-107]. Furthermore, it is already stated by several guidelines that any person with a BMI above 40, or a BMI higher than 35 with comorbidities, should be advised to undergo bariatric surgery [108-110] since it has proven to resolve obesity-related comorbidities like diabetes, hypertension, sleep apnoea, and asthma and reduces mortality rates. An issue of concern, however, is whether an ESRD-patient is fit enough to undergo a risk reducing operation with the risk of complications in itself. In general, the complication and mortality rates after bariatric surgery have declined greatly over the years to about 0.3% [111]. A few studies on bariatric surgery in ESRD-patients show low complication and 90-day mortality rates close to 0% [15,16,112-114]. This is important to acknowledge because survival of patients on dialysis is far worse compared to the survival after RT. [18] Therefore, every possible RT candidate should be carefully assessed to see if possible complications of bariatric surgery, although being very low, would not pose a risk for the transplantation. In our opinion, every obese recipient should be informed about this possibility, being aware of possible risks. A clinical trial is ongoing to investigate whether bariatric surgery before RT has benefits (ClinicalTrials.gov, number NCT01913392).

### Limitations

It has to be acknowledged that a systematic review and meta-analysis can only be as good as the quality of the included studies. Potentially, several types of bias have been introduced in this analysis. The individual studies are prone to sampling bias because of the fact that they are observational studies. It is possible that, due to publication bias, the results have become skewed. However, based on funnel plot analyses (data not shown), we can safely state that publication bias is minimal. Another limitation is that not all studies have clearly specified the definition of certain outcome measures. For example, not all studies mention whether or not cases of acute rejection are, in fact, biopsy proven or the used definition of DGF. This may introduce bias in the analyses leading to heterogeneity. Moreover, only a few studies defined whether the transplanted kidneys were from live or deceased donors (donation after circulatory death or donation after brain death), which is a confounding factor in the pooled analysis of DGF. It would be interesting to have this specific information, to see whether the hypothesis that high BMI recipients have better outcome when receiving a kidney from a living donor or a standard criteria ‘donation after brain death’ donor kidney can be confirmed. In line of this limitation, also the ‘pre-transplant’ status of a recipient is of importance; whether he or she was transplanted pre-emptively or was on haemo- or peritoneal dialysis prior to transplantation has an impact on the outcome after RT.

It would be interesting if future studies would include other parameters that take into account the fat distribution of a recipient, as the BMI does not; for example, the waist circumference or hip-waist-ratio [115]. It could be that outcome would change if these parameters were correlated to outcome of RT recipients.

## Conclusions

Based on our results, we make the following recommendations:

- RT candidates should not be excluded for transplantation on the basis of BMI alone.
- High BMI renal transplant candidates should be referred to high-volume/tertiary referral centres in order to keep knowledge about these category of patients centralized.
- Informed consent procedures for obese RT candidates should include the risk profiles associated with obesity and RT outcome.
- Both patients and clinicians should be aware of the importance of weight loss prior to transplantation.
- (Morbidly) obese RT candidates should be informed about all possible weight reduction methods, ranging from dietary restriction under supervision of a dietician to the option of bariatric surgery.
  - ○ Obese RT candidates with a BMI between 30 and 35 should be referred to conventional methods of weight reduction, with help of a dietician.
  - ○ Obese RT candidates with a BMI >35 and comorbidities or a BMI >40 should be referred for bariatric surgery, based on bariatric guidelines. Based on our experience, this could pose some difficulties for dialysis patients because of the required diet prior to bariatric surgery and age limits for bariatric surgery. Some bariatric centres have a maximum age limit of 60 for bariatric surgery. However, a large part of ESRD patients is over 60 years old. For these reasons, in our opinion, ESRD patients with morbid obesity cannot be compared to ‘regular’ morbid obese individuals.
- Ideally, in high BMI RT candidates, the process of weight reduction should be initiated several years before RT to ensure an adequate time period for remedial measures to become effective.
- Despite the poorer outcome of RT in these patients, the survival benefit of RT over dialysis needs to be emphasized. Therefore, we need to maximize our efforts for obese ESRD patients to get access to RT, and develop strategies to reduce the risks associated with RT in this patient category.
- Innovations in surgical techniques should be stimulated. For example, robot-assisted techniques for implantation could be promising for this specific patient category [116].

## Additional file

Additional file 1:
**Search strings.**

## Abbreviations

BMI: Body mass index
CI: Confidence interval
CMV: Cytomegalovirus
ESRD: End-stage renal disease
GRADE: Grades of Recommendation, Assessment, Development and Evaluation
KDIGO: Kidney Disease Improving Global Outcomes
NODAT: New onset diabetes after transplantation
OR: Odds ratio
PRISMA: Preferred Reporting Items for Systematic Reviews and Meta-Analyses
RR: Risk ratio
RT: Renal transplantation
