# Erratum: Body mass index and outcome in renal transplant recipients: a systematic review and meta-analysis

**DOI:** 10.1186/s12916-015-0387-3

**Published:** 2015-06-15

**Authors:** Jeffrey A Lafranca, Jan NM IJzermans, Michiel GH Betjes, Frank JMF Dor

**Affiliations:** Department of Surgery, division of HPB & Transplant Surgery, Erasmus MC, University Medical Center Rotterdam, ‘s Gravendijkwal 230, PO BOX 2040, 3000 CA Rotterdam, The Netherlands; Department of Nephrology, Erasmus MC, University Medical Center Rotterdam, ‘s Gravendijkwal 230, PO BOX 2040, 3000 CA Rotterdam, The Netherlands

## Abstract

This is an Erratum to *BMC Medicine* 2015, 13:111 indicating the correct name for one of the authors.

Please see related article: http://www.biomedcentral.com/1741-7015/13/111

## Erratum

Authors’ corrected note:

Jan NM IJzermans last name was incorrectly spelled in the author list and was indicated as Jan NM IJermans in our published article [1]. The erratum includes this author’s correct last name. The erratum includes this information.

Corrected text:

Jan NM IJzermans
