# Supplementary material for: Body mass index and outcome in renal transplant recipients: a systematic review and meta-analysis
Source: BMC Med. 2015 May 12;13:111. doi: 10.1186/s12916-015-0340-5 (PMC4427990; doi:10.1186/s12916-015-0340-5)
Supplement: Additional file 1: — Search strings. [file 12916_2015_340_MOESM1_ESM.docx]

**SUPPLEMENTAL DATA 1**

**Embase 4614**

('kidney transplantation'/exp OR 'kidney donor'/de OR ((kidney* OR renal) NEAR/3 (transplant* OR donor* OR donation*)):ab,ti) AND ('body mass'/de OR 'body weight'/de OR 'body weight disorder'/de OR 'weight gain'/de OR obesity/exp OR ((body NEXT/1 (mass OR weight)) OR BMI OR quetelet OR obes* OR overweight*):ab,ti) AND ('graft survival'/de OR 'graft dysfunction'/exp OR 'postoperative complication'/de OR 'anastomosis dehiscence'/de OR 'wound infection'/de OR 'postoperative infection'/de OR risk/exp OR 'follow up'/de OR (((surviv* OR function* OR dysfunction* OR failure* OR reject* OR delay* OR loss OR result*) NEAR/3 (graft* OR transplant*)) OR ((postoperat* OR postsurg* OR 'post operative' OR 'post surgical' OR posttransplant* OR 'post transplantation' OR wound*) NEAR/3 (complicat* OR infect*)) OR (anastomo* NEXT/1 dehiscence) OR transplantectom* OR outcome* OR complicat* OR (follow* NEXT/1 up) OR risk*):ab,ti)

**Medline OvidSP 2192**

("kidney transplantation"/ OR ((kidney* OR renal) ADJ3 (transplant* OR donor* OR donation*)).ab,ti.) AND ("Body Mass Index"/ OR exp "body weight"/ OR ((body ADJ (mass OR weight)) OR BMI OR quetelet OR obes* OR overweight*).ab,ti.) AND ("graft survival"/ OR "Primary Graft Dysfunction"/ OR exp "postoperative complication"/ OR exp "wound infection"/ OR exp risk/ OR "Follow-Up Studies"/ OR (((surviv* OR function* OR dysfunction* OR failure* OR reject* OR delay* OR loss OR result*) ADJ3 (graft* OR transplant*)) OR ((postoperat* OR postsurg* OR "post operative" OR "post surgical" OR posttransplant* OR "post transplantation" OR wound*) ADJ3 (complicat* OR infect*)) OR (anastomo* ADJ dehiscence) OR transplantectom* OR outcome* OR complicat* OR (follow* ADJ up) OR risk*).ab,ti.)

**Cochrane central 84**

(((kidney* OR renal) NEAR/3 (transplant* OR donor* OR donation*)):ab,ti) AND (((body NEXT/1 (mass OR weight)) OR BMI OR quetelet OR obes* OR overweight*):ab,ti) AND ((((surviv* OR function* OR dysfunction* OR failure* OR reject* OR delay* OR loss OR result*) NEAR/3 (graft* OR transplant*)) OR ((postoperat* OR postsurg* OR 'post operative' OR 'post surgical' OR posttransplant* OR 'post transplantation' OR wound*) NEAR/3 (complicat* OR infect*)) OR (anastomo* NEXT/1 dehiscence) OR transplantectom* OR outcome* OR complicat* OR (follow* NEXT/1 up) OR risk*):ab,ti)

**Web of science 1940**

TS=((((kidney* OR renal) NEAR/3 (transplant* OR donor* OR donation*))) AND (((body NEAR/1 (mass OR weight)) OR BMI OR quetelet OR obes* OR overweight*)) AND ((((surviv* OR function* OR dysfunction* OR failure* OR reject* OR delay* OR loss OR result*) NEAR/3 (graft* OR transplant*)) OR ((postoperat* OR postsurg* OR "post operative" OR "post surgical" OR posttransplant* OR "post transplantation" OR wound*) NEAR/3 (complicat* OR infect*)) OR (anastomo* NEAR/1 dehiscence) OR transplantectom* OR outcome* OR complicat* OR (follow* NEAR/1 up) OR risk*)))

**PubMed publisher 42**

(((kidney*[tiab] OR renal[tiab]) AND (transplant*[tiab] OR donor*[tiab] OR donation*[tiab]))) AND ((body mass*[tiab] OR body weight*[tiab] OR BMI[tiab] OR quetelet[tiab] OR obes*[tiab] OR overweight*[tiab])) AND ((((surviv*[tiab] OR function*[tiab] OR dysfunction*[tiab] OR failure*[tiab] OR reject*[tiab] OR delay*[tiab] OR loss[tiab] OR result*[tiab]) AND (graft*[tiab] OR transplant*[tiab])) OR ((postoperat*[tiab] OR postsurg*[tiab] OR post operative*[tiab] OR post surgical*[tiab] OR posttransplant*[tiab] OR post transplant*[tiab] OR wound*[tiab]) AND (complicat*[tiab] OR infect*[tiab])) OR anastomotic dehiscence*[tiab] OR transplantectom*[tiab] OR outcome*[tiab] OR complicat*[tiab] OR follow up*[tiab] OR risk*[tiab])) AND publisher[sb]

**Google scholar 200**

"kidney|renal transplantation|donor|donation" "body mass|weight"|"weight gain"|obesity|BMI|overweight survival|dysfunction|complication|complications|dehiscence|infection|"follow up"|followup
